# Physicochemical, Sensory, and Nutritional Properties of Foods Affected by Processing and Storage Series II

**DOI:** 10.3390/foods13010156

**Published:** 2024-01-02

**Authors:** Sidonia Martínez, Javier Carballo

**Affiliations:** Food Technology Area, Faculty of Sciences, University of Vigo, 32004 Ourense, Spain; carbatec@uvigo.es

Food processing has several different purposes. First, it aims to increase the shelf life of foods by protecting them from physical, chemical, and biological agents of deterioration or to sanitize them by destroying the pathogens that can be present in raw materials and compromise consumer health. However, nowadays, consumers are increasingly demanding and better informed, and they are concerned with the quality of the foods they consume and the consequent effects on their health; they also demand new, sensorially surprising foods with new aromas, flavors, and textures. This new challenge facing food technology to incorporate new ingredients and additives to obtain more nutritive, safe, and surprising foods occupies a good part of the efforts of the current food industry. On the other hand, food storage is a necessary practice due to the seasonal nature of the production of some foods or the need to ensure their supply in other cases. 

Food processing and storage are therefore normal and necessary activities to obtain and enjoy the wide range of foods that we know, retaining their safety and diverse sensory and nutritional profiles. However, sometimes these processes have negative effects that result in a decrease in nutritional value, in an alteration of sensory properties, or even in a generation of compounds that can be harmful to the health of the consumer. To avoid undesirable effects and obtain increasingly nutritious foods that meet the needs and expectations of consumers, it is necessary to constantly generate knowledge about the effects of technological treatments and storage on food components and properties to optimize these processes and minimize their negative effects.

In a previous Special Issue devoted to this same topic [1], 19 contributions were collected, including different studies on food canning, dehydration, fermentation, irradiation, marinating, and cooking, as well as on the use of preservatives and the effect of storage. This new Special Issue is a continuation of the previous one and presents new advances in and approaches to food processing and storage, the optimization of processes, and the incorporation of new components that allow the valorization of materials and the production new foods, therefore adding value to the food industry.

Heat treatment is one of the oldest and most effective procedures used to reduce the activity of microorganisms and enzymes and prolong the shelf life of foods. The effectiveness of heat treatment and its effects on the organoleptic characteristics of foods are conditioned by the environment in which it is applied. Daei et al. studied the effects of two mild thermal treatments (63 °C or 40 °C for 3 min) and in two different packaging media (brine with different NaCl concentrations or a vinegar solution) on some of the physicochemical (weight loss, phenolic compounds, ascorbic acid, and firmness) and microbiological (microbial load) characteristics of truffles (*Terfezia claveryi*) during 160 days of storage. A control of unheated truffles was also studied. Rthe authors showed that both methods were effective in reducing the microbial load. The 63 °C treatment in vinegar reduced the weight loss and microbial spoilage and increased the firmness of truffles during storage. However, the contents of antioxidant compounds (phenolics and ascorbic acid) decreased as the intensity of heat treatment increased. Despite the loss of antioxidant activity, treatment at 63 °C for 3 min in a vinegar solution proved effective in increasing the shelf life of truffles without appreciable damage in quality attributes.

Canning (heat treatment after packaging in hermetic containers) is a classical process used for food preservation. After the initial study by Appert [2], researchers have not stopped trying to optimize the treatments, packaging materials, and filling media. Marine foods are typically canned, resulting in high quality fish and seafood, which is appreciated by consumers. The chemical components and sensory attributes of marine foods are differently affected by canning; accordingly, widely varying effects of canning have been reported. In this Special Issue, two manuscripts offer valuable contributions to this topic. Gómez-Limia et al. evaluated the effect of a filling medium (sunflower oil, olive, or spicy olive oil) on the color (CieLab parameters) and sensory characteristics of European eel (*Anguilla anguilla*) for different steps of the canning process (after frying, canning, and 2 and 12 months of storage). The authors found significant color changes during the process both in the fish and in the filling medium They reported that the color changed the most in eel packed in olive oil during sterilization, and spicy olive oil was the filling medium that experienced the largest color changes, probably because of the migration of some spice components into the oil during canning and storage. The sensory characteristics of the eel were also influenced by the filling medium, with the eels packed in sunflower oil receiving the highest consumer scores. Biogenic amines are important in canned seafoods as quality indicators and because of their harmful effects on health. Qu et al. studied the effect of the storage (temperature and time) on the biogenic amine content of five canned seafood species (mud carp, sardine, mantis shrimp, scallop, and oyster). The levels of nine biogenic amines (histamine (HIS), phenylethylamine (PHE), tyramine (TYM), putrescine (PUT), cadaverine (CAD), tryptamine (TRY), spermine (SPM), spermidine (SPD), and octopamine (OCT)) were analyzed in these canned seafoods along 12 months of storage at different temperatures (4, 10, 25, and 30 °C). The authors detected HIS, PHE, PUT, and TYM in 100% of the samples; CAD, SPM, and SPD were detected in 60%; and OCT was detected in 40% of the samples. Additionally, they observed that the recommended maximum limits of HYS an TYR were exceeded in the canned mud carp and scallop when stored at 30 °C. They concluded that low storage temperatures inhibit the production of biogenic amine in canned seafood.

Pulsed electric field (PEF) is an emerging and promising technology used for destroying microorganisms at low temperatures in a continuous flow regime [3]. Extensive research efforts have recently been devoted to the use of this technology to replace others that are more harmful to the organoleptic characteristics of foods or to their safety. Delso et al. [4] studied the use of PEF to decontaminate red wine, avoiding the use more problematic classical techniques such as the addition of sulfur dioxide or the sterilizing filtration. The authors evaluated the suitable of PEF for the decontamination of red wine after alcoholic (elimination of *Saccharomyces cerevisiae*) and malolactic (elimination of *Oenococcus oeni*) fermentation. Their results showed that the populations of these two microbial species could be reduced up to 4.0 log10 cycles by applying PEF, and sublethal damage and synergistic effects with SO_2_ were observed. After 4 months, wine treated with this technology after alcoholic fermentation lacked viable yeasts, whereas that treated after malolactic fermentation and 20 ppm of SO_2_ contained less than 100 CFU/mL of *O. oeni*. No detrimental effects on the oenological or sensory parameters of treated wines were observed after storage.

Cooking is possibly the oldest of the procedures used for preparing food for consumption. Freeze-drying is an almost perfect dehydration method because the elimination of water in situ via sublimation maintains the structure of the food, favoring its rehydration and the conservation of textures, flavors, and aromas. Cambero et al. studied the textural properties, sensory quality, and rehydration kinetics of three Spanish chickpea varieties to identify the most suitable cooking conditions to obtain high-quality freeze-dried chickpeas for use in the preparation of a traditional Spanish dish called *cocido*. The authors also evaluated the post-rehydration sensory quality of various vegetables and meat portions (the other constituents of this dish), which were cooked under different conditions and freeze-dried. The results show that by selecting the appropriate cooking and freeze-drying conditions for the ingredients. the sensory quality of this traditional Spanish dish could be reproduced after rehydration of the freeze-dried components with water, heating in a microwave oven for 5 min, and letting them rest for 10 min.

Food storage is, in most cases, a necessary operation because some foods are seasonally produced; in other cases, adapting the pattern of production to those of consumption is difficult. Some food preservation procedures (i.e., canning, dehydration, and irradiation) destroy or inhibit the biological agents that cause spoilage and allow the long-term storage of food. However, such preservation procedures modify the organoleptic properties of foods. Some foods should therefore be stored in the fresh state, and other strategies to minimize their alteration during storage must be adopted. Such is the case of pomegranate, a fruit that has recently received attention due to its healthy components. The shelf life of this fruit ranges from 3–4 months when stored in air to 4–6 months when stored in a controlled atmosphere. One of the alterations that can appear during storage and that limit the shelf life of this fruit is husk scald (HS). This is a superficial brown discoloration that is restricted to the husk and does not affect the interior of the fruit but substantially limits its acceptance by consumers. Maghoumi et al. reviewed the factors involved in the development of HS, the modes of action of these factors, and their association with postharvest treatments. The authors discussed a hypothesis regarding the etiology and mechanism of development of HS in view of research on this subject; they also considered the postharvest treatments proposed for its control and the possible targets of these treatments. 

Tomato is another fruit which is preferably consumed fresh and that is how it should be kept. It is a climacteric fruit that typically has as shelf life of 7–11 days when stored at room temperature. To extend the shelf life and maintain the quality it is crucial to control the processes of respiration and transpiration, as well as microbial contamination. To achieve these objectives, the application of edible coatings has emerged as an alternative in recent years. In this special issue, Flores-López et al. evaluated the effect of an alginate/chitosan nanomultilayer coating without (NM) and with *Aloe vera* liquid fraction (NM+Av) on the postharvest quality of tomato fruit stored at 20 °C and 85% relative humidity. Authors reported that both nanomultilayer coatings had comparable effects on firmness and pH values. However, the NM+Av coating significantly reduced weight loss and molds and yeasts counts compared to uncoated fruit. Moreover, it notably lowered O_2_ consumption and CO_2_ production, also inhibiting ethylene synthesis. The usefulness of the NM+Av coating was confirmed by visual evaluation and instrumental color measurement. In view of their results, authors concluded that the NM+Av coating is a potential alternative to improve tomato preservation.

Freezing followed by frozen storage is the most successful procedure for long-term storage, producing after-thawing products that differ minimally from the fresh products. However, for optimal results, the freezing, storage, and thawing conditions for each product must be correctly adjusted. Wang et al. studied the effects of two different storage temperatures (−18 °C and −55 °C) for 180 days of storage on three different parts (naked body, big belly, and middle belly) of bluefin tuna. The effects were evaluated through determining the tissues’ water-holding capacity, malondialdehyde content, color differences, salt-soluble protein content, free amino acid contents, endogenous fluorescent protein content, and water distribution and migration. The authors found minimal quality changes during short-term storage, but storage at −55 °C significantly improved the tuna quality parameters compared with those achieved with storage at −18 °C. The results showed that the water-holding capacity, salt-soluble protein content, and water content of tuna decreased as the storage time increased. The authors concluded that because the tuna quality changed little during short-term frozen storage, a storage temperature of −18 °C is suitable for short-term storage prior to sale.

As indicated at the beginning of this Editorial, consumers are becoming increasingly concerned about the quality of the foods they consume and their effects on health. At the same time, they are also demanding and attracted to new foods with different and surprising organoleptic properties. This demand has forced the food industry to strengthen its efforts to incorporate new ingredients and additives and to develop new manufacturing processes capable of generating different aromas, flavors, and textures. To overcome such challenges, in-depth research is necessary to clarify the effects of new ingredients, additives, and processes and of their interactions, as well as to develop analytical methods and quality control procedures to monitor these new products. The last five articles contained in this special issue are devoted to this research topic. The textural attributes of cooked rice are the dominant indicators of eating quality. Their appropriate assessment is therefore critical to obtain a satisfactory product. Kaewsorn et al. evaluated the precision and sensitivity of a back-extrusion (BE) test to assess the texture of cooked germinated brown rice (GBR) during the production process. They studied the effects of different soaking and incubation durations on the hardness, toughness, and stickiness of Khao Dawk Mali 105 GBR, noting the high precision of the BE test in the measurement of these properties, as indicated by the repeatability and reproducibility results. The sensitivity was also satisfactory, as indicated by the high coefficient of variation of the texture properties. 

Rice is also widely used as source of starch, having several different destinations in the food industry. Starch gels have many uses, such as for the development of gluten-free starch-based products. To improve the gel structure and stability of the rice starch (RS) used for this purpose, Xu et al. studied the effect of the addition of guar gum and locust bean gum to rice starch gel on its pasting, rheological properties, and freeze–thaw stability. Analyses were performed with a rapid viscosity analyzer, rheometer, and texture analyzer. The authors found that both gums can modify the pasting properties, as indicated by increases in the peak, trough, and final viscosities, thereby preventing the short-term tendency of the retrogradation shown by RS. The measurements of dynamic viscoelasticity also showed that the starch–gum system exhibited improved viscoelastic properties (higher storage modulus (G’) values) compared with those of starch alone. Compared with the control system, the hysteresis loop area of the systems containing guar gum or locust bean gum was reduced by 37.7% and 24.2%, respectively, indicating that the addition of gum could enhance the shear resistance and structure recovery properties. The thermodynamic properties showed that both gums retarded the short- and long-term retrogradations of the RS gels. The addition of galactomannans significantly improved the textural properties and freeze–thaw stability of the RS gel, with guar gum being more effective than locust bean gum, which could be attributed to the differences in the mannose-to-galactose ratio. The results of this study provide alternatives for gluten-free recipes with improved freeze–thaw stability and texture properties. 

During legume processing, by-products with applications in the food industry are generated. Among these is aquafaba, the liquid obtained after cooking legumes. Fuentes Choya et al. studied the composition (total solid, protein, fat, and carbohydrate contents) and culinary properties (foaming and emulsifying capacities, and foam and emulsion stabilities) of *Pedrosillano* (a traditional Spanish variety) chickpea aquafaba produced with different cooking liquids (water, vegetable broth, meat broth, and the filling liquid from canned chickpeas). Then, using these aquafaba types as ingredients, they prepared and studied French egg-free baked meringues, using meringues containing egg white as control. The sensory characteristics of the meringues were analyzed using instrumental and panel-tester techniques. The authors found that the nature of the cooking liquid and the intensity of the heat treatment affected the composition and culinary properties of the aquafaba, which showed appropriate foaming and intermediate emulsifying capacities in all cases, with the aquafaba prepared using the filling medium of commercial canned chickpeas producing the meringue the most similar to that produced with egg white in terms of performance. Regarding the meringue characteristics, the aquafaba-based products had fewer alveoli, higher hardness and fracturability, and minimal color changes after baking compared with those manufactured using egg white. Those produced with aquafaba prepared using meat and vegetable broths were rated the lowest by a panel of testers. 

Veganism is increasing among people, and new products are constantly being developed to satisfy vegan needs and preferences [4]. Mihaylova et al. reported the development of healthy vegan bonbons enriched with lyophilized peach powder. They prepared three different formulations containing 10%, 20%, and 30% lyophilized peach power (LPP). The bonbons immediately after manufacturing were analyzed in terms of physical (size, weight, moisture, ash, color, water activity, texture, and microscopic imagery), microbiological (total aerobic mesophiles, and mold and yeast counts), and nutritional (contents in protein, fat, sugars, fiber, monounsaturated fats, omega-3 fatty acids, energy) characteristics, as well as in terms of parameters indicating antioxidant properties (antioxidant activity, and flavonoid and phenolic contents). Analyses of texture and water activity were repeated after 5 days, and microbiological analyses were repeated after 3 and 5 days of storage. All the bonbons had the same weight, size, and ash content. The addition of LPP, however, decreased the protein, sugar, fat, omega-3 fatty acid, energy, and total phenolic contents, but increased the monounsaturated fatty acid and total flavonoid contents. The addition of LPP reduced the antioxidant activity and increased the hardness, fracturability, and adhesiveness of the bonbons, whereas color was not affected. Regarding the microbial load, the addition of LPPC inhibited the microbial growth during storage. The results of this study show the possibility of using lyophilized peach powder as an ingredient for the manufacture of heathy raw vegan bonbons.

Plant-based bioactive compounds (BCs) and their incorporation into processed foods have attracted attention from researchers and the food industry due being perceived as safer and healthier by consumers. In the last paper in this Special Issue, Vilas-Boas et al. compared the composition, antioxidant activity, and antimicrobial properties of polyphenol-rich extracts obtained from *Lavandula pedunculata* (LP), a native Portuguese species, using maceration (CE), or microwave-assisted (MAE) or ultrasound-assisted (UAE) extraction. The authors found that rosmarinic acid (58.68–48.27 mg/g dry extract) and salvianolic acid B (43.19–40.09 mg/g DE) were the representative phenolic compounds in the extracts. They also reported that the three methods allowed them to obtain extracts with high antioxidant activity, highlighting the ORAC results (1306.0 to 1765.5 mg Trolox equivalents (TE)/g DE). The extracts obtained using MAE and CE showed outstanding growth inhibition of *Bacillus cereus*, *Staphylococcus aureus*, *Escherichia coli*, *Salmonella enterica*, and *Pseudomonas aeruginosa* (>50%, at 10 mg/mL). The MAE extract had the lowest IC_50_ (0.98 mg DE/mL) for the inhibition of angiotensin-converting enzyme and the highest IC_50_ for the inhibition of α-glucosidase and tyrosinase (87 and 73% of inhibition, respectively, at 5 mg/mL). Moreover, these extracts were safe for caco-2 intestinal cells, where no mutagenic effects were detected. UAE was less efficient in obtaining LP polyphenol-rich extracts. The efficiency of MAE equaled that of CE, saving time and energy. From these results, the authors concluded that LP shows potential as a sustainable raw material, allowing diverse extraction methods to be used to safely obtain health-promoting food and nutraceutical ingredients.
